# Many correlates of poor quality of life among substance users entering treatment are not addiction-specific

**DOI:** 10.1186/s12955-016-0439-1

**Published:** 2016-03-03

**Authors:** Ashley E. Muller, Svetlana Skurtveit, Thomas Clausen

**Affiliations:** Norwegian Centre for Addiction Research, Institute of Clinical Medicine, University of Oslo, Postbox 1171 Blindern, 0318 Oslo, Norway; Department of Pharmacoepidemiology, Division of Epidemiology, The Norwegian Institute of Public Health, Oslo, Norway; Addiction Unit, Sørlandet Hospital HF, Kristiansand, Norway; Fulbright Scholar (2014-15), Alcohol Research Group (ARG), Emeryville, CA USA

**Keywords:** Substance use disorder, Quality of life, Exercise, Social support, Physical well-being

## Abstract

**Background:**

Quality of life (QoL) is an important measure and outcome within chronic disease management and treatment, including substance use disorders (SUD). The aim of this paper was to investigate correlates of poorer QoL of individuals entering SUD treatment in Norway, in order to identify subgroups that may most benefit from different interventions.

**Methods:**

Twenty-one treatment facilities invited all incoming patients to participate. Five hundred forty-nine patients who enrolled between December 2012 and April 2015 are analyzed. QoL, substance use, mental and physical comorbidities, and exercise behaviors were measured. Multinomial regression analysis was used to determine variables significantly associated with poorer QoL.

**Results:**

The majority of both genders (75 %) reported “poor” or “very poor” QoL at intake. Depression showed a strong association with poor QoL (relative risk ratio [RRR] 3.3, 95 % confidence interval [CI] 1.0–10.3) and very poor QoL (RRR 3.8, 1.2–11.8) among women. Physical inactivity among men was associated with very poor QoL (RRR 2.0, 1.1–3.7), as was reporting eating most meals alone (RRR 2.6, 1.4–4.8). Evaluating one’s weight as too low was also associated with poor QoL (RRR 2.0, 1.0-3.9) and very poor QoL (RRR 2.0, 1.1–3.7) among men. Consuming methadone/buprenorphine was a protective factor for men reporting poor QoL (RRR 0.5, 0.3–0.9) and very poor QoL (RRR 0.4, 0.2–0.9), as well as for women reporting very poor QoL (RRR 0.2, 0.0–0.6).

**Conclusions:**

Factors associated with poorer QoL among other healthy and clinical populations, such as impaired social and physical well-being and psychological distress, were also seen associated in this sample. Treatment should be targeted towards patients with these particular vulnerabilities in addition to focusing on substance-related factors, and interventions proven to improve the QoL of other populations with these vulnerabilities should be explored in a SUD context.

## Background

Treatments for chronic diseases are increasingly evaluated through subjective patient assessments on outcomes other than morbidity and mortality, and quality of life (QoL) stands out as the most common and a relevant measurement. QoL is a subjective appraisal of a patient’s life in that instant: how satisfied the patient is with their current physical health, mental health, social relationships, and environment, to use the World Health Organization’s four emphasized domains [1]. The substance use disorder (SUD) treatment field has until recently less systematically collected and prioritized the QoL of patients, in comparison with other medical fields [2]. Importantly, QoL measures add patients’ subjective assessments of the impacts that SUD and its treatment can have on their lives [3]. Such measures privilege the patient in determining whether their employment status, health, and family contact, for example, are satisfactory or not. QoL measures may also help clinicians recognize problems other than the specifics of the disorder and hence make better treatment decisions and priorities (see Frisch et al 1994 [4] and Foster et al 1999 [5] for discussions).

Individuals with SUD typically report significantly poorer QoL than the general population and as low as those with other serious psychiatric disorders [5–8]. Poor QoL may also be a predictor of treatment readiness; two qualitative studies have shown that the desire to redress the negative effects of a SUD on a patient’s life and improve their QoL is a more explicit goal of treatment among patients than is reducing substance use itself [9, 10]. Interestingly, poor QoL has not been consistently predicted by SUD-specific characteristics, such as the types of substances used, frequencies of use, and lengths of problematic usage [7, 11]. Yet QoL plays a role in recovery from a SUD: Laudet et al. found that higher QoL at treatment discharge predicted abstinence better than traditional SUD characteristics [9]. In addition to measuring QoL for its own sake, as a subjective measure of functioning [12], exploring and addressing the dissatisfaction with various life domains that impair QoL may improve abstinence outcomes and help patients access a fuller range of health benefits after treatment.

Few consistent predictors of QoL have been found, speaking to the global nature of this measure. However, good mental health appears to be a strong protective factor among opioid dependents, alcohol dependents, and polysubstance users [11, 13, 14], dual-diagnosis patients [15], and non-SUD populations [16], perhaps because of the far-reaching effects that psychiatric symptoms and disorders exert on an individual’s life. Along with better mental health, exercise can also have an enhancing effect on QoL among healthy and clinical, non-SUD populations [17–20]. Trials with the SUD population measuring QoL after exercise are lacking, and the mechanisms by which exercise affects QoL are still unknown. A reduction of comorbid risk factors [21], mediation of the deleterious effect of chronic physical conditions on QoL [22, 23], and improved self-efficacy and other psychological constructs [24] have been suggested as mechanisms. Interest in exercise overall among the SUD population has led to evidence suggesting exercise plays a role in improving abstinence rates and other substance outcomes such as craving, decreasing depressive and anxious symptoms, and improving physical condition (see Zschucke et al 2012 [25] for a review).

The interaction of the social environment with the development of a SUD and treatment trajectory have been of interest since the 1980’s, and the nuances of social factors progressively explored. For example, the detrimental effects of a substance-using network on abstinence appear to outweigh both the quantitative and qualitative support received in that network [26, 27]. Furthermore, the composure and impact of social networks appears to differ between genders; substance use among partners and family members has stronger negative effects on women’s treatment outcomes than on men’s [28–30]. Women and men also have different factors, including social variables, which set them at risk for relapse or treatment drop-out [27]. Less research has been devoted to social factors’ impact on QoL, despite low QoL being another drop-out risk factor [9], and this relationship does not seem to have been investigated with a gender lens. In this article, we therefore describe the QoL of persons presenting for SUD treatment, and investigate gender differences in substance, health, and social factors associated with poorer QoL. More knowledge of these factors, which are also determinants of successful treatment outcomes, will be able to guide treatment providers towards improved and targeted clinical practices.

## Methods

### Participants and setting

Cross-sectional data were drawn from the Norwegian cohort of patient in opioid maintenance treatment study (NorComt), a multi-center study involving 21 treatment facilities throughout Norway [31]. Facilities provided inpatient and/or outpatient treatment, both opioid maintenance treatment and medication-free. Data were collected 2012–2015 from patients when they entered facilities, via structured, face-to-face interviews by facility staff which took an average of 90 min. The research team trained facility staff in use of the questionnaires through a series of group workshops and interview guides. The only inclusion criterion was admittance into a substance use disorder treatment facility within the past twelve weeks, regardless of primary substance type(s). A total of 1416 patients were identified as potentially relevant for this study. Mainly due to logistical difficulties at the facilities, 318 patients were not informed about the study or invited to participate, resulting in their ineligibility. Seventy-four percent of the 746 eligible patients agreed to participate, while 129 declined and 68 either did not respond or did not meet for the interview. In total, 549 participants enrolled from the 21 facilities.

### Measures

Overall QoL was measured by a single item asking, “how would you rate your quality of life?” [31]. Respondents answered using a five-point Likert-type scale from “very poor” to “very good.” This question is the same as the global measure included in the World Health Organization’s brief and full QoL tools (WHOQOL-BREF and WHOQOL-100, respectively) [1, 32], selected for its discrete time burden and reliability with multiple-item QoL scales [33]. As a generic question validated among a variety of healthy and clinical groups, including in Norway [34, 35], it enables the comparison of patients’ subjective evaluations of their lives, made while embedded within their particular environmental contexts [1].

The interview questionnaire also included excerpts from the EuropASI, a validated version of the Addiction Severity Index adapted for European use [36], to collect substance variables and information on social networks. Patients selected substances from a list of 18 that included, among others, alcohol, cannabis, heroin, and methadone/buprenorphine. The two subscores of the Hopkins Symptoms Checklist-25 were used to assess mental health. A subscore above 1.0 on the 10-item anxiety subscore indicated symptom levels of clinical anxiety, while a subscore above 1.0 on the 15-item depression subscore indicated clinical depression [37]. Regular physical activity was defined as more than twice weekly over the past six months. In addition, dichotomous sociodemographic independent variables included civil status (“single” or “married/partnered”), Norwegian-born, unemployed, educational attainment of primary school or less, and having at least one child. “With whom do you eat most of your meals?” was used as a proxy for social contact [38, 39], and dichotomized into “alone” or, if patients selected friends, families, or others, “with others”.

### Analysis

Bivariate analyses tested the individual association of each independent variable with each gender’s overall QoL and between each gender, using chi-squares and t-tests or Mann-U Whitney tests, as appropriate and according to distribution. Independent variables were selected based on previous research into relevant QoL-determinants among SUD patients and new variables of interest. These variables included: treatment type (opioid maintenance treatment or medication-free inpatient), most recently used substance by type, comorbid mental illness (depression or anxiety), chronic somatic illness (including cancer, chronic obstructory pulmonary disease, and HIV), self-reported physical inactivity and satisfaction with one’s weight, social network variables, and sociodemographic variables.

We performed multinomial logistic regression analysis for each gender including independent variables with a relationship (p < 0.1) to QoL in bivariate analysis, and age was treated as a continuous covariate. As the distribution of scores did not meet the stringent requirements of ordinal logistic regression, the five-point Likert scale of “very poor”, “poor”, “neither good nor poor”, “good”, and “very good” was collapsed into three categories (“very poor”, “poor”, and “neutral/good/very good”) to enable multinomial logistic regression. As the majority of patients reported “poor” or “very poor” QoL, these categories were analyzed separately – against the reference category of “neutral/good/very good” – in order to determine which variables were associated with the worst-off. The outcome measure was the relative risk ratio (RRR) with confidence interval using the category neutral/good/very good as the reference.

SPSS version 22 was used for all statistics.

## Results

### Sample descriptives

As displayed in Table 1, this heterogeneous sample was comprised of 156 women (28 %) and 393 men (72 %), with an average age of 33.7 (*SD* 9.9) Most reported being single (93.4 %), unemployed (87.2 %), and having less than a secondary education (59.4 %). Symptoms of clinical anxiety (56.7 %) were common, as were symptoms of clinical depression (56.2 %) and additional somatic chronic illnesses. Regular physical activity was reported by 41.7 % of participants, and about half said they were dissatisfied with their current weight (54.4 %). The majority were polysubstance users (91.5 %) and had previous SUD treatment experience (92.3 %). The most common substance used in the past six months was heroin (by 24.4 % of participants), followed by OMT medication (21.5 %), amphetamines (19.6 %), cannabis (16.9 %), and alcohol (7.9 %). About half said their social network was mainly comprised of other substance users (50.5 %), compared to one-third who reported a substance-free network (32.2 %) and 17.1 % reporting no network at all. Spending mealtimes alone was reported by 45.9 %, while 25.8 % ate with family, 16.9 % with friends, and 11.1 % with others.

**Table 1:** Sample descriptives of incoming substance use disorder patients in Norway, 2012–2015

	n (%)
Age (mean)	33.7 (*SD* 9.9)
Female	156 (28.4)
Single	512 (93.3)
Norwegian-born	496 (90.7)
Primary education or less	323 (59.4)
Unemployed	479 (87.2)
Has children	245 (45.6)
Clinical anxiety symptoms	303 (56.7)
Clinical depression symptoms	300 (56.2)
Chronic disease(s)	398 (72.5)
Physically inactive	319 (58.3)
Physically active	228 (41.7)
Weight evaluation	
Satisfied with weight	235 (45.6)
Considers self underweight	126 (24.5)
Considers self overweight	154 (29.9)
Medication-free treatment	266 (48.5)
OMT	283 (51.5)
Injected, past 4 weeks.	196 (35.8)
Substance user category	
Prescribed OMT and/or medications only	8 (1.5)
Substance- and medication-free	4 (0.7)
Single-substance user	34 (6.3)
Polysubstance user	497 (91.5)
Previous SUD treatment in months, median	
Inpatient	8.0
Outpatient	13.5
Most used substance, past 6 months	
Alcohol	43 (7.9)
Cannabis	92 (16.9)
Heroin	133 (24.4)
Methadone/buprenorphine	120 (21.5)
Benzodiazepine	38 (7.0)
Amphetamine	107 (19.6)
Social network	
Substance-free	176 (32.2)
Substance-using	277 (50.6)
Socially isolated	94 (17.2)
Stable recent living situation	428 (78.5)
Eats most meals alone	245 (45.9)
East with someone else	289 (54.1)
Recent living situation	
Homeless	56 (10.2)
Temporary housing	26 (4.7)
Institution	127 (23.2)
Owned or rented	267 (48.7)
Other	72 (13.1)

*SUD* substance use disorder*,* OMT *opioid maintenance treatment*

### Current overall QoL and other characteristics by gender

**Fig. 1:**
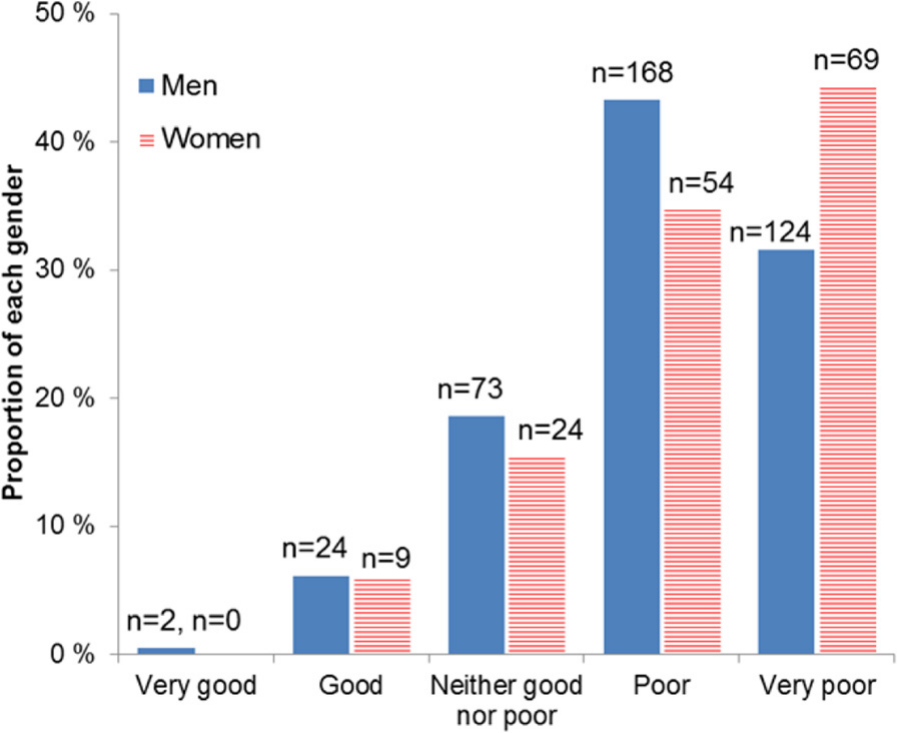
Quality of life distribution of incoming substance use disorder patients in Norway, 2012–2015. Detailed legend: Quality of life was skewed towards negative ratings among this sample of entering Norewgian substance use disorder patients. Seventy-five pecent rated their quality of life as “poor” or “very poor”, and distributions did not vary by gender

Men and women’s ratings of their overall QoL skewed towards poorer ratings, as seen in Fig. 1. Approximately three-fourths of both genders self-reported their QoL as “very poor” or “poor”, and 25 % rated it “neutral” or higher. There were no significant differences in the distribution of women and men’s QoL.

### Correlates of poor overall QoL by gender

The distribution of QoL (in three categories) according to independent variables is displayed in Table 2. Among both genders reporting very poor QoL, the majority also reported symptoms of clinical anxiety (68.9 % of men and 69.1 % of women) and depressive symptoms (64.8 and 70.6 %, respectively). Both men and women who reported methadone/buprenorphine as their “most-used substance” were more likely to report good, neutral, or very good QoL (33.0 and 31.3 %, respectively) than very poor QoL (18.0 and 7.2 %).

**Table 2:** Overall quality of life (QoL) of patients entering substance use disorder treatment in Norway, 2012–2015

	Men’s overall QoL			Women’s overall QoL		
	very good/good/neutral *n* = 99	poor *n* = 168	very poor *n* = 124	very good/good/neutral *n* = 33	poor *n* = 54	very poor *n* = 69
	n (%)	n (%)	n (%)	n (%)	n (%)	n (%)
Age (mean (SD))	34.3 (10.3)	33.4 (9.1)	32.3 (9.6)	31.6 (10.9)	35.4 (11.1)	33.8 (10.5)
Single	90 (91.8)	157 (95.2)	117 (95.1)	30 (93.8)	48 (90.6)	63 (92.6)
Norwegian-born	86 (87.8)	149 (91.4)	117 (95.1)	30 (93.8)	48 (90.6)	58 (84.1)
Primary education or less	56 (57.7)	94 (57.3)	77 (62.6)	19 (59.4)	32 (61.5)	41 (61.2)
Unemployed	85 (87.6)	138 (85.7)	113 (93.4)	27 (84.4)	48 (90.6)	62 (91.2)
Has children	47 (50.5)	70 (42.7)	48 (40.0)	18 (58.1)	26 (50.0)	30 (44.1)
Clinical anxiety symptoms	**48** (49.0)*	**80** **(49.7)**	**84** **(68.9)**	**13** (41.9)*	**30** **(56.6)**	**47** **(69.1)**
Clinical depression symptoms	**44** (44.9)*	**84** **(52.2)**	**79** **(64.8)**	**11** (35.5)*	**33** **(62.3)**	**48** **(70.6)**
Chronic disease(s)	71 (72.4)	113 (68.5)	91 (74.0)	26 (81.3)	42 (79.2)	53 (76.8)
Physically inactive	**44** (44.9)*	**99** **(60.4)**	**76** **(61.8)**	20 (62.5)	21 (39.6)	25 (36.2)
Physically active	**54** **(55.1)**	**65** **(39.6)**	**47** **(38.2)**	12 (37.5)	4 (8.2)	9 (14.8)
Weight evaluation						
Satisfied with weight	**57** **(61.3)** ^**a**^	**67** **(43.2)**	**52** **(44.4)**	17 (53.1)	17 (34.7)	22 (36.1)
Considers self underweight	**21** **(22.6)**	**48** **(31.0)**	**38** **(32.5)**	4 (12.5)	4 (8.2)	9 (14.8)
Considers self overweight	**15** **(16.1)**	**40** **(25.8)**	**27** **(23.1)**	11 (34.4)	28 (57.1)	30 (49.2)
Medication-free treatment	42 (42.9)	80 (48.5)	68 (55.3)	12 (37.5)	23 (43.4)	39 (56.5)
OMT	56 (57.1)	85 (51.5)	55 (44.7)	20 (62.5)	30 (56.6)	30 (43.5)
Injected, past 4 weeks.	37 (37.8)	55 (33.5)	44 (36.1)	11 (34.4)	16 (30.2)	29 (42.0)
Substance user category						
Prescribed OMT and/or medications only	4 (4.1)	1 (0.6)	1 (0.8)	**2** **(6.3)** ^**a**^	**0** **(0.0)**	**0** **(0.0)**
Substance- and medication-free	1 (1.0)	3 (1.8)	0 (0.0)	**0** **(0.0)**	**0** **(0.0)**	**0** **(0.0)**
Single-substance user	7 (7.2)	12 (7.4)	6 (5.0)	**3** **(9.4)**	**2** **(3.8)**	**4** **(5.8)**
Polysubstance user	85 (87.6)	147 (90.2)	113 (94.2)	**27** **(84.4)**	**51** **(96.2)**	**65** **(94.2)**
Previous SUD treatment in months, median						
Inpatient	6.0	4.0	5.0	10.5	4.0	5.5
Outpatient	4.0	6.0	9.0	13.0	12.5	12.0
Most used substance, past 6 months						
Alcohol	7 (7.1)	9 (5.5)	7 (5.7)	2 (6.3)	6 (11.3)	12 (17.4)
Cannabis	14 (14.3)	37 (22.4)	27 (22.0)	2 (6.3)	6 (11.3)	5 (7.2)
Heroin	21 (21.6)	34 (20.9)	30 (24.6)	10 (31.3)	15 (28.3)	19 (27.5)
Methadone/buprenorphine	**32** (33.0)*	**37** **(22.7)**	**22** **(18.0)**	**10** (31.3)**	**12** **(22.6)**	**5** **(7.2)**
Benzodiazepine	6 (6.1)	10 (6.1)	7 (5.7)	1 (3.1)	6 (11.3)	7 (10.1)
Stimulant	16 (16.3)	33 (20.0)	28 (22.8)	7 (21.9)	7 (13.2)	21 (30.4)
Social network						
Substance-free	**38** **(38.8)** ^**a**^	**64** **(38.8)**	**31** **(25.2)**	14 (45.2)	12 (22.6)	14 (20.6)
Substance-using	**45** **(45.9)**	**76** **(46.1)**	**61** **(49.6)**	14 (45.2)	32 (60.4)	45 (66.2)
Socially isolated	**15** **(15.3)**	**25** **(15.2)**	**31** **(25.2)**	3 (9.7)	9 (17.0)	9 (13.2)
Stable recent living situation	76 (77.6)	132 (81.0)	91 (75.2)	28 (87.5)	43 (81.1)	49 (71.0)
Eats most meals alone	**36** (38.7)**	**76** **(47.2)**	**76** **(63.3)**	6 (19.4)	18 (34.0)	30 (44.1)
East with someone else	**57** **(61.3)**	**85** **(52.8)**	**44** **(36.7)**	25 (80.6)	35 (66.0)	38 (55.9)
Recent living situation, past 4 weeks						
Homeless	12 (12.2)	16 (9.7)	15 (12.3)	1 (3.1)	1 (1.9)	11 (15.9)
Temporary housing	5 (5.1)	2 (1.2)	8 (6.6)	4 (12.5)	2 (3.8)	5 (7.2)
Institution	25 (25.5)	44 (26.7)	26 (21.3)	7 (21.9)	10 (18.9)	14 (20.3)
Owned or rented	47 (48.0)	80 (48.5)	60 (49.2)	15 (46.9)	30 (56.6)	29 (42.0)
Other	9 (9.2)	23 (13.9)	13 (10.7)	5 (15.6)	10 (18.9)	10 (14.5)

Variables approaching significance are in bold text. ^a^ *p* < 0.1; *p < 0.05; **p < 0.01

*QoL* Quality of life*, OMT* opioid maintenance treatment*,SUD* substance use disorder

Most men who reported good, neutral, or very good QoL were physically active (55.1 %), and most reported being satisfied with their weight (61.3 %). While men with a substance-using network represented about half of each QoL category (45.9 % of those with good, neutral, or very good QoL; 46.1 % of those with poor QoL; and 49.6 % of those with very poor QoL), socially isolated men were represented more in the very poor QoL category (25.2 %) and men with a substance-free network were represented more in the very good, good, or neutral QoL category (38.8 %). Finally, 63.3 % of men with very poor QoL also said they ate most of their meals alone, while 61.1 % of men reporting the highest category of QoL ate with others.

Table 3 below displays the association of variables, selected for their significant bivariate relationships to QoL displayed in Table 2, with reporting poor and very poor QoL. In the final multinomial logistic regression, experiencing symptoms of clinical depression was strongly associated with poor QoL (RRR 3.3, CI 1.0–10.3) and very poor QoL (RRR 3.8, CI 1.2–11.8) among women.

**Table 3:** Unadjusted and adjusted relative risk ratios (RRR) of reporting poor or very poor quality of life (QoL) by selected health, social well-being, and substance-specific indicators

	Men		Women	
	Unadjusted RRR (%CI)	Adjusted RRR (%CI)	Unadjusted RRR (%CI)	Adjusted RRR (%CI)
Poor QoL vs. good/neutral QoL				
Age	1.0 (1.0–1.0)	1.0 (1.0–1.0)	1.0 (1.0–1.0)	1.0 (1.0–1.1)
Clinical anxiety symptoms	1.0 (0.6–1.7)	0.7 (0.4–1.3)	1.8 (0.8–4.4)	0.9 (0.3–2.7)
Clinical depression symptoms	1.3 (0.7–2.2)	1.4 (0.7–2.6)	3.0 (1.2–7.5)**	3.3 (1.0–10.3)*
Physically inactive	1.9 (1.1–3.1)**	**1.6 (0.9–2.8)** ^**a**^	-----	-----
Weight evaluation (reference: satisfied)				
Considers self underweight	2.0 (1.1–3.6)**	2.0 (1.0–3.9)*	-----	-----
Considers self overweight	2.2 (1.1–4.5)**	**2.0 (0.9–3.9)** ^**a**^	-----	-----
Eats meals alone	1.4 (0.8–2.3)	1.5 (0.9–2.7)	-----	-----
Social network (reference: no network)			-----	-----
Substance-free	1.0 (0.5–2.2)	1.5 (0.6–3.4)	-----	-----
Substance-using	1.0 (0.5–2.1)	1.1 (0.5–2.6)	-----	-----
Substance user category (reference: polysubstance user)				
Prescribed OMT and/or medications only	-----	-----	0.0 (-)^b^	-----
Substance- and medication-free	-----	-----	0.0 (-)^b^	-----
Single-substance user	-----	-----	0.4 (0.1–2.2)	-----
Most used substance: methadone/buprenorphine	**0.6 (0.3–1.0)** ^**a**^	0.5 (0.3–0.9)*	0.4 (0.2–1.8)	0.6 (0.2–1.7)
Very poor QoL vs. good/neutral QoL				
Age	1.0 (1.0–1.0)	1.0 (0.9–1.0)	1.0 (0.9–1.0)	1.0 (1.0–1.1)
Clinical anxiety symptoms	2.3 (1.3–4.0)**	1.4 (0.7–2.9)	3.1 (1.3–7.5)**	1.3 (0.4–3.8)
Clinical depression symptoms	2.3 (1.3–3.9)**	1.5 (0.8–3.1)	4.4 (1.8–10.8)**	3.8 (1.2–11.8)*
Physically inactive	2.0 (1.6–3.4)**	2.0 (1.1–3.7)*		
Weight evaluation (reference: satisfied)				
Considers self underweight	**2.0 (1.0–3.8)**	2.0 (1.1–3.7)*	-----	-----
Considers self overweight	2.0 (1.0–4.1)	1.6 (0.7–3.6)	-----	-----
Eats meals alone	2.7 (1.6–4.8)***	2.6 (1.4–4.8)*	-----	-----
Social network (reference: no network)				
Substance-free	0.4 (0.2–0.9)**	0.7 (0.3–1.7)	-----	-----
Substance-using	0.7 (0.3–1.3)	0.9 (0.4–2.1)	-----	-----
Substance user category (reference: polysubstance user)				
Prescribed OMT and/or medications only	-----	-----	0.0 (-)^b^	-----
Substance- and medication-free	-----	-----	0.0 (-)^b^	-----
Single-substance user	-----	-----	0.6 (0.1–2.6)	-----
Most used substance: methadone/buprenorphine	0.5 (0.2–0.8)*	0.4 (0.2–0.9)*	0.2 (0.0–0.6)*	0.2 (0.0–0.6)*

Variables approaching significance are in bold text. ^a^ *p* < 0.1; *p < 0.05; **p < 0.01; ***p < 0.001. ^b^Too few persons to calculate RRR were in this category

*QoL* Quality of life, *OMT* opioid maintenance treatment

Physical inactivity had an association to reporting very poor QoL (RRR 2.0, CI: 1.1–3.7) for men, and judging their weight to be inappropriately low was associated with both poor QoL (RRR 2.0, CI: 1.0–3.9) and very poor QoL (RRR 2.0, CI: 1.1–3.7). The trend towards poor QoL among men who viewed their weight as too high (RRR 2.0, CI: 0.9–3.9) and who were not physically active (RRR 1.6, CI: 0.9–2.8) continued, although no longer significant (*p* = 0.09 for both variables). Eating meals alone continued to be a risk factor for reporting very poor QoL (RRR 2.6, CI: 1.4–4.8) among men.

Consuming methadone/buprenorphine had a protective effect against reporting very poor QoL for men (RRR 0.4, CI: 0.2–0.9) and for women (RRR 0.2, CI: 0.0–0.6), and a protective effect against reporting poor QoL for men (RRR 0.5, CI: 0.3–0.9).

## Discussion

This analysis of incoming SUD patients in Norway shows that physical, mental and social well-being measures were of more consequence to QoL than SUD-specific indicators, with the exception of a strong protective effect of using methadone or buprenorphine. After adjusting for age and other significant variables, depressive symptoms increase women’s risk of reporting poorer QoL. Men who were not physically active more often reported poorer QoL, as did those who considered themselves underweight and those who ate most of their meals alone (a sign of limited social networks). These variables indicate vulnerabilities at treatment entry which may warrant additional attention through targeted mental health, physical health, and social support interventions, and which also contextualize SUD patients as persons susceptible to many of the same health and social concerns as other clinical and healthy populations, in addition to experiencing the serious effects of a SUD.

The significantly impaired QoL ratings of our sample were consistent with other studies [2], and our sample again shows poorer QoL than among other clinical groups [40]. The correlation of mental health problems with low QoL is also an established finding in the literature among chronic disease patients and the general population as well as among the SUD population [11, 13, 14], including in Norway [41]. The use of methadone or buprenorphine before entering treatment was associated with reporting better QoL, and their use was the only SUD-related indicator with a significant association to QoL [7]. This may reflect that these individuals were more settled, in that they did not need to “chase” illicit short-acting opiates on the street or buy from risky markets, and hence experienced more stabilized lives. Methadone and buprenorphine are prescribed in OMT in Norway, and while some of these individuals may not have been receiving formal prescription of OMT medications, use of such medicines still had positive effects on their QoL.

Few large studies have explored SUD patients’ QoL in connection with other physical well-being indicators, including exercise behavior, and our data show that being physically active improves the likelihood that male patients report higher QoL. Exercising behavior is frequently found to correlate with better QoL among other chronic disease populations and to improve QoL [19, 20]. This should prompt the SUD research field to utilize existing exercise research and QoL research from non-SUD groups when developing evidence-based treatment options, as well as to measure and exploit the potential for exercise to be used as a QoL-boosting activity within treatment. Exercise should be integrated into treatment for the very reason that this population presents with such impaired QoL. One smaller study suggests that significant QoL improvements can be seen even after modest doses of exercise [42].

As with physical inactivity, men’s concerns with broader physical self-perceptions have been negatively correlated with quality of life among other populations. For example, Pope et al. found that feeling too lean or insufficiently muscular is particularly dissatisfying for university-aged men, and we see impaired QoL among both genders in healthy and clinical groups when preoccupied with weight and other negative body images [43–45].

“Eating alone” as a risk factor for men’s very poor QoL also points to the importance of normalizing social relationships and increasing social contact. Spending mealtimes alone can indicate low social participation and limited networks among the elderly [46], particularly for men [38], and social exclusion has been found to be correlated with poorer QoL among psychiatric patients [47]. Again, the social well-being needs of SUD patients may not be so different from other chronic disease sufferers or from the non-clinical population. Many studies have highlighted the importance of partners, families, and other elements of social settings on women’s initiation and maintenance of substance use [27, 29], and our data reminds us that social exclusion can still be a source of vulnerability for men with SUD.

Another main finding is that the negative effects of poor physical, mental, and social well-being are a pattern across this heterogeneous sample. In fact, these indicators were more decisive to our sample’s QoL than having a chronic physical condition, typical protective demographic factors such as being employed [48], and even addiction severity measures such as polysubstance use and recent injecting behavior. This finding is most clearly seen among men, where physical inactivity and a lack of social contact have a clear relationship to the worst QoL. The absence of many SUD-specific correlates shows that physical and social well-being factors, perhaps less commonly considered relevant to SUD patients by their treatment staff, are in fact burdensome and should be addressed more specifically as part of treatment. Specifically, it is important to give patients chances and support to prioritize their own needs and gain support for positive activities in order to address their bodily and social concerns.

Accessing QoL at intake can be an opportunity to learn about patient vulnerabilities which may not be uncovered through more objective questioning of various pre-determined domains, or a focus limited to substance use patterns. Our findings also support the continued measurement of QoL during treatment to guide further treatment plans as well as to be an outcome measure of treatment, which for a chronic condition must be monitored and addressed during the course of the disorder, at various phases, inclusive of during treatment [49]. Knowing the variables that influence patients’ well-being can help target treatment toward patient-identified goals, and such patient improvement may improve treatment engagement, retention and success. If treatment’s goal of recovery and improved well-being is to be achieved, services must be offered on multiple levels and empower patients to improve numerous areas of their life, without focusing only on substance-use outcomes [50, 51]. One such tool may be opportunities and support for physical activity as well as developing supportive social networks. Indeed, group-based exercise as part of treatment would likely improve both physical and social outcomes and improve QoL both directly and indirectly, thereby enhancing recovery.

Several limitations must be recognized when interpreting these results. The cross-sectional nature of this analysis prohibits drawing conclusions of causation, but the associations we found support further emphasis on improving physical and social well-being in treatment, including through investment in longitudinal studies to assess exercise’s effect on the QoL of the SUD population specifically. There may be other significant but unmeasured associations that confounded the relationships we found to QoL; for example, body mass index may explain the correlations between physical inactivity and poor QoL for men. Future studies should include larger sample sizes to thoroughly explore factors which may differentially impact women and men. The 74 % of patients who agreed to participate may not be representative of the entire incoming SUD patient population and there may have been inadvertent selection mechanisms at inclusion. However, characteristics of this sample were similar to Norwegian patient register data [37], and including patients up to twelve weeks after starting treatment prevented the de facto exclusion of patients in the worst shape. We therefore consider our estimates to be conservative rather than too large. The study involved a large geographic sample of treatment facilities, and it is to the study’s benefit that the sample provides such heterogeneity with regards to substance-related characteristics.

## Conclusion

The characteristics that we found to be associated with poorer quality of life in this sample of substance use disorder patients – reporting depressive symptoms (for women); being physically inactive, dissatisfied with one’s physical self, and reporting some element of social isolation (for men) – are vulnerabilities that are not unique to this population. Not only do these findings highlight the importance of addressing mental health and of providing support for physical and social well-being during treatment, but they serve as a reminder that SUD patients are vulnerable to many of the same situations and conditions as those without a SUD. Treatment should therefore take care to not lose sight of such factors by prioritizing directly substance-related issues, often externally determined to be more important. Measuring quality of life, throughout the treatment trajectory, recognizes patients as sources of such important and otherwise uncollectable information.

### Ethics

The study was approved by The Regional Committee for Research Ethics in Norway (REK 2012/1131). Written informed consent was obtained from all study participants.
